# Factors Associated with Trust in Public Authorities Among Adults in Norway, United Kingdom, United States, and Australia Two Years after the COVID-19 Outbreak

**DOI:** 10.3389/ijph.2023.1605846

**Published:** 2023-08-02

**Authors:** Daicia Price, Tore Bonsaksen, Janni Leung, Caitlin McClure-Thomas, Mary Ruffolo, Gary Lamph, Isaac Kabelenga, Amy Ostertun Geirdal

**Affiliations:** ^1^ School of Social Work, University of Michigan, Ann Arbor, MI, United States; ^2^ Department of Health and Nursing Science, Faculty of Social and Health Sciences, Inland Norway University of Applied Sciences, Elverum, Norway; ^3^ Department of Health, Faculty of Health Studies, VID Specialized University, Stavanger, Norway; ^4^ Faculty of Health and Behavioural Sciences, The University of Queensland, Brisbane, QLD, Australia; ^5^ Department of Health Social Care and Medicine, Edge Hill University, Ormskirk, United Kingdom; ^6^ Department of Social Work and Sociology, School of Humanities and Social Sciences, University of Zambia, Lusaka, Zambia; ^7^ Department of Social Work, Child Welfare and Social Policy, Faculty of Health Sciences, Oslo Metropolitan University, Oslo, Norway

**Keywords:** coronavirus, pandemic, vaccination, trust, public authorities

## Abstract

**Objectives:** This study aimed to examine the levels of trust in information provided by public authorities 2 years after the COVID-19 outbreak and to examine factors associated with trust.

**Methods:** Using a cross-national approach, online survey data was collected from four Western countries—Australia, Norway, the United Kingdom, and the United States of America. Differences in reports of very low or low levels of trust were examined by age, gender, area of residence, and the highest level of education in the four countries.

**Results:** Levels of trust in the public authorities’ information were highest among Norwegian respondents and lowest among U.K. respondents. Lower levels of trust in public authorities were found among males, individuals living in rural or remote areas, and those with lower levels of education.

**Conclusion:** The outcomes contribute to knowledge regarding differences between socio-demographic groups and countries regarding the levels of trust people have in public authorities’ information concerning a crisis, such as COVID-19. Strategies to promote trust in societies in different countries could consider these socio-demographic differences.

## Introduction

The coronavirus (COVID-19) was declared a global pandemic on 11 March 2020, as media outlets shared information about a virus spreading rapidly and having fatal outcomes. Following the acknowledgment of a public health crisis, questions and concerns about the potential responses of public authorities to protect public health and safety surfaced across various forms of media [1–4].

As public authorities across the globe began to respond to the emerging pandemic, disparities among approaches were observed [1]. Each country determined its policies and procedures for health, safety, and fiscal management [1, 5, 6]. Governmental entities in Western countries responded similarly, including limiting travel, requiring face coverings, and closing businesses and schools. Despite the similar preventive measures implemented globally, compliance levels and sentiments toward the implementation differed between countries. Citizen responses to these actions varied from frustration to relief. While some individuals felt confident in the decisions of government entities to implement restrictions and follow mandates, others described a lack of trust in the information provided and beliefs that the government had hidden motives [2, 7].

Trust in government officials is developed and maintained by delivering accurate information, having consistency in responses, transparency in challenges and decision-making, and producing positive outcomes for citizens by providing valuable resources to address identified needs [4, 7, 8]. Theories of trust and social capital offer a framework to examine the connection between trust and engagement in recommendations related to a public health crisis. The theory of social capital highlights the interdependence of trust and a society that can function effectively [9]. Relationships between the general civilian public and officials with a responsibility to deliver safety and security are critical during healthcare emergencies to increase positive attitudes and behaviors from citizens that support compliance with recommendations and enhance the social efficacy of public health and wellness [10, 11].

Trust is needed to incorporate self-efficacy in the most effective and efficient ways during any stressful situation [9, 12]. People’s trust in the medical advice and information received from the government about COVID-19 had an impact on the public’s compliance with mandated lockdowns and safety recommendations [6, 7, 13–18]. Without trust, the likelihood of individuals making independent decisions to adhere to safety recommendations, even during a health crisis, is reduced. During a global crisis, the level of control a person feels they have over their situation can adversely impact their ability to engage in behaviors that are identified as helpful [7, 10, 17–19]. Associations between rates of trust in public authorities and cooperation from citizens have been noted during previous crises [9, 20]. The impact of trust in public authorities has been magnified during and after the COVID-19 pandemic as individuals are encouraged to participate in prevention strategies such as minimizing contact with others, wearing masks, and obtaining a vaccine to prevent the rapid spread of the virus [2, 21, 22].

Studies examining trust in public authorities during COVID-19 have provided information about ongoing connections between trust and outcomes. Goldfinch et al. (2021) found that levels of trust in public authorities were associated with how the government responded to the pandemic. If the responses of the government were viewed to be effective in reducing the spread and lethality of the coronavirus, trust levels were increased, and the adverse impacts of COVID-19 were less than in areas where rates of trust were lower [5, 17, 22]. This is consistent with previous public health issues reported [16, 23–25]. In a cross-national study nine months after the COVID-19 outbreak, identifying as female, having higher levels of education, and living in urban areas were associated with trust in public authorities; however, being infected with the coronavirus was associated with distrust [26].

Researchers note that the U.S. and the U. K. had higher levels of infection and fatalities and citizens were more likely to report distrust in the responsiveness of public authorities to COVID-19 [7, 14, 19, 24, 26]. A focus on supporting the national economy and not the health and safety of citizens, along with perceived slow and disjointed responses at the federal and state or provincial levels to respond to the global pandemic, has been noted as a contributor to the lack of trust in public authorities [2, 5, 7, 17, 19, 27].

As COVID-19 continues to be a leading cause of death globally, with 6,897,025 deaths as of 11 April 2023, ongoing interventions are critical (WHO, 2022). Coronavirus has highlighted disparities between confidence and trust in public authorities. The inconsistencies require ongoing knowledge contribution to examine population responses in countries with similar social-political contexts [28]. As citizens worldwide vary in their willingness to engage in COVID-19 prevention and intervention measures, understanding levels of trust in public authorities is critical in the ongoing efforts to address the continuous disruption that COVID-19 has on individual lives and communities.

To contribute to the scientific knowledge, examining trust in public authorities can provide information for public authorities to engage citizens unwilling or hesitant to adhere to policy, procedure, and practice recommendations to reduce the spread and lethality of COVID-19 at various time intervals of an international health crisis. The primary source of information specific to COVID-19 often relies on information provided by public authorities [25]. Increasing the availability of independent findings based on individual reports is critical when considering levels of trust in public authorities to offer additional references for knowledge.

To expand on existing knowledge, this study examines the levels of trust in information provided by public authorities two years after the COVID-19 outbreak in four Western countries—Australia, Norway, the United Kingdom, and the United States—to examine factors associated with trust.

## Methods

### Design and Procedures

This study reports from the third cross-sectional survey disseminated openly in four countries (Norway, U.K., U.S., and Australia) during the COVID-19 pandemic. Participants were recruited from the countries where the researchers were based. The current survey was available for the general public’s participation between November 2021 and January 2022. A public landing site for the web-based survey was established and shared through personal and professional networks using electronic mail communication and various social media platforms (e.g., Facebook ads; university Facebook and Twitter websites). The initiator of the project was AØG from OsloMet. Due to ethical considerations and permissions, each country had its own project lead. The survey was simultaneously co-developed by the researchers in two languages—Norwegian and English. It was based on previous surveys conducted by the research group in the early phase (April 2020) and mid-point (November 2020) of the pandemic outbreak [26, 29–32]. Language and cultural differences were considered during the survey development process. During this period, access to COVID-19 vaccinations was available in each country where participants were recruited, and many public health restrictions were lifted. While the participants were recruited from four different countries, the survey captured the perspectives of individuals in those countries who completed the survey and is not intended to be representative of the country’s population.

### Inclusion

To be included in the study, participants had to be 18 years or older, understand Norwegian or English, live in Norway, USA, U.K., or Australia, and have access to an electronic device and internet.

### Measures

#### Sociodemographic Characteristics

The sociodemographic variables include age group (categories measured were: 18–29 years, 30–39 years, 40–49 years, 50–59 years, 60–69 years, 70 years and above), gender identity (male, female, other, prefer not to respond), place of residence (rural or farming area; town or suburb; city), and highest completed education level (high school or associated/technical degree or lower, Bachelor’s degree, Master’s/doctoral degree).

#### Trust in Public Authorities

The primary outcome variable—trust in communications issued by authorities—was constructed from the following question: “What is your level of trust in the information provided to the public by government and public authorities about the COVID-19 pandemic?” Participants were asked to respond using a 5-point scale with options of very low, low, moderate, high, or very high.

### Statistical Analysis

Descriptive statistics were examined for each variable by country, and chi-square tests were conducted to examine differences. Responses to the level of trust were analyzed as (1) very low or low, and (2) moderate, high, or very high. Proportions of participants reporting very low or low trust were cross-tabulated by the socio-demographic variables with chi-squared tests to examine the bi-variate associations. Multivariate logistic regression was conducted with all socio-demographic variables included on low or very low levels of trust as the outcome (reference: moderate, high, or very high trust). Odds ratios with 95% confidence intervals (CI) were presented with *p*-values. First, a regression was conducted, testing for all main effects. Second, the regression analysis was repeated, including interaction terms of each socio-demographic variable by country, to test for the robustness of effects by country. For following up significant country interactions, stratified regression analyses (separate analysis for each country) were conducted, and odds ratios of the socio-demographic variables on trust that had a significant by-country interaction were presented. There was no missing data.

### Ethics

The data collected in this study was anonymous. The researchers adhered to all relevant regulations in their respective countries concerning ethics and data protection.

## Results

### Participants

The total number of participants was 1,649, with 242 (14.7%) from Norway, 255 (15.15%) from the U.K., 915 (55.5%) from the U.S., and 237 (14.4%) from Australia (see Table 1). The age distribution shows a spread of ages, skewing towards younger ages in the U.K. and USA samples and older ages in the Australian sample. Women comprised the larger part of the sample (75% identified as female, 20% identified as male, and 4% identified as other/prefer not to respond), particularly in the Australian sample (81% female). The majority of the sample from Norway and Australia lived in city areas, while the majority of the sample from the U.K. and USA were in towns or suburbs. Over 70% of participants had a bachelor’s degree or higher; the Australian sample had the highest proportion of individuals with vocational training, and the USA sample had the highest proportion with a Master’s degree or above.

**TABLE 1:** Descriptive Statistics (Factors Associated with Trust in Public Authorities Among Adults in Norway, UK, US, and Australia Two Years after the COVID-19 Outbreak, Norway, United Kingdom, United States, and Australia. 2022).

	OverallN = 1,649		NorwayN = 255		U.K.N = 255		USAN = 915		AustraliaN = 237		Chi-sq
	n	%	n	%	n	%	n	%	n	%	*p*
Age Group											
18–29	269	16.3	33	13.6	34	13.3	181	19.8	21	8.9	<0.001
30–39	425	25.8	58	24.0	56	22.0	296	32.3	15	6.3	
40–49	458	27.8	51	21.1	76	29.8	304	33.2	27	11.4	
50–59	256	15.5	59	24.4	67	26.3	76	8.3	54	22.8	
60–69	157	9.5	30	12.4	20	7.8	37	4.0	70	29.5	
70 and above	84	5.1	11	4.5	2	0.8	21	2.3	50	21.1	
Gender											
Female	1,242	75.3	188	77.7	198	77.6	664	72.6	192	81.0	0.001
Male	336	20.4	50	20.7	53	20.8	194	21.2	39	16.5	
Other/prefer not to say	71	4.3	4	1.7	4	1.6	57	6.2	6	2.5	
Living Area											
Rural/remote area	249	15.1	21	8.7	45	17.6	179	19.6	4	1.7	<0.001
Town/suburb	815	49.4	86	35.5	159	62.4	510	55.7	60	25.3	
City/metropolitan area	585	35.5	135	55.8	51	20.0	226	24.7	173	73.0	
Highest Education											
Secondary/high school or below	157	9.5	32	13.2	17	6.7	83	9.1	25	10.5	<0.001
Vocational training/associate degree	234	14.2	19	7.9	47	18.4	112	12.2	56	23.6	
Bachelor’s degree	575	34.9	92	38.0	86	33.7	312	34.1	85	35.9	
Master’s degree or above	683	41.4	99	40.9	105	41.2	408	44.6	71	30.0	
Trust Information by authorities											
Very low or low	445	27.0	38	15.7	82	32.2	265	29.0	60	25.3	<0.001
Moderate, high, or very high	1,204	73.0	204	84.3	173	67.8	650	71.0	177	74.7	

In our overall sample, 27% reported very low or low trust. The U.K. had the highest proportion reporting very low or low trust (32%), followed by the USA (29%), Australia (25%), and Norway with the lowest proportion (16%), *p* < 0.001.

### Trust in Sample Subgroups

Significant group differences in the level of trust in information from public authorities are shown in Table 2. Significant differences (≤0.001) were observed in reports of trust in information between countries, age groups, genders, living areas, and educational levels. Self-reports of very low or low trust were more common in the 40–49 age group, male or other genders, individuals living in rural or remote areas, and those with lower levels of education.

**TABLE 2:** Cross-tabulation of trust by socio-demographic factors and adjusted regression on trust (Factors Associated with Trust in Public Authorities Among Adults in Norway, UK, US, and Australia Two Years after the COVID-19 Outbreak, Norway, United Kingdom, United States, and Australia. 2022).

	Bi-Variate Analysis			Multi-Variate Analysis	
	Trust by socio-demographic subgroups (n [%])			Adjusted logistic regression of very low or low trust (ref: moderate, high or very high)	
	Very low or low	Moderate, high, or very high	*p*	OR [95%CI]	*p*
Country					
Norway	38 [15.7%]	204 [84.3%]	<0.001	1.00 [ref]	
U.K.	82 [32.2%]	173 [67.8%]		2.30 [1.45–3.66]	<0.001
USA	265 [29.0%]	650 [71.0%]		1.96 [1.30–2.95]	0.001
Australia	60 [25.3%]	177 [74.7%]		2.12 [1.29–3.49]	0.003
Age Group					
18–29	48 [17.8%]	221 [82.2%]	<0.001	0.58 [0.31–1.11]	0.101
30–39	112 [26.4%]	313 [73.6%]		1.25 [0.68–2.29]	0.470
40–49	154 [33.6%]	304 [66.4%]		1.75 [0.97–3.17]	0.065
50–59	69 [27.0%]	187 [73.0%]		1.35 [0.73–2.48]	0.339
60–69	40 [25.5%]	117 [74.5%]		1.19 [0.63–2.25]	0.600
70 and above	22 [26.2%]	62 [73.8%]		1.00 [ref]	
Gender					
Female	269 [21.7%]	973 [78.3%]	<0.001	1.00 [ref]	
Male	142 [42.3%]	194 [57.7%]		2.75 [2.11–3.60]	<0.001
Other/prefer not to say	34 [47.9%]	37 [52.1%]		3.39 [2.02–5.68]	<0.001
Living Area					
Rural/remote area	91 [36.5%]	158 [63.5%]	<0.001	1.83 [1.26–2.64]	0.001
Town/suburb	235 [28.8%]	580 [71.2%]		1.39 [1.05–1.85]	0.023
City/metropolitan area	119 [20.3%]	466 [79.7%]		1.00 [ref]	
Highest Education					
Secondary/high school or below	59 [37.6%]	98 [62.4%]	<0.001	2.73 [1.82–4.10]	<0.001
Vocational training/associate degree	87 [37.2%]	147 [62.8%]		2.02 [1.43–2.85]	<0.001
Bachelor’s degree	155 [27.0%]	420 [73.0%]		1.43 [1.08–1.88]	0.011
Master’s degree or above	144 [21.1%]	539 [78.9%]		1.00 [ref]	

### Regression of Socio-Demographic Factors Associated With Trust

Factors associated with trust in public authorities’ information are shown in Table 2. Levels of trust in information varied by country (*p* < 0.001); the odds of reporting very low or low trust were about two times higher in the U.K., USA, and Australia, compared to Norway. In the unadjusted analysis, the 40–49 age group had higher odds of reporting very low or low trust than the 70+ group. Still, it was not statistically significant after controlling for all other variables examined. After adjustment for all variables, males and those who selected “other” or “preferred not to say” had 2.75 [2.11–3.60] and 3.39 [2.02–5.68] higher odds of reporting very low or low trust (*p* < 0.001) than females, respectively. Area of residence was associated with trust, with those living in rural or remote areas and those living in towns or suburbs more likely to report low or very low trust than those living in city or metropolitan areas. A dose-response trend was observed with education, such that the lower the level of education, the higher odds of reporting very low or low trust.

Significant interactions were observed for gender*country and education*country. Stratified regression analyses by country show that the dose-response trend of lower education with lower trust was only observed in the USA (see Table 3). In the U.K., the lowest education group had higher odds of low or very low trust, but it was not statistically significant. In Australia, the lowest education group had the lowest odds of low trust (*p* < 0.001). For the Norway participants, only the lowest education group had higher odds of very low or low trust compared to the highest education group (*p* = 0.003).

**TABLE 3:** Associations of Education and Gender on Very Low or Low Trust by Country (Factors Associated with Trust in Public Authorities Among Adults in Norway, UK, US, and Australia Two Years after the COVID-19 Outbreak, Norway, United Kingdom, United States, and Australia. 2022).

	Stratified analyses on very low or low trust (ref: moderate, high, or very high trust)							
	Norway		U.K.		USA		Australia	
	OR [95% CI]	*p*	OR [95% CI]	*p*	OR [95% CI]	*p*	OR [95% CI]	*p*
Education by Country								
Secondary/high school or below	7.04 [1.92–25.76]	0.003	1.90 [0.64–5.62]	0.249	3.54 [2.03–6.18]	<0.001	0.09 [0.03–0.28]	<0.001
Vocational training/associate degree	1.73 [0.40–7.39]	0.461	0.66 [0.30–1.49]	0.321	3.13 [1.94–5.07]	<0.001	1.52 [0.46–4.97]	0.492
Bachelor’s degree	2.75 [0.97–7.81]	0.057	0.95 [0.51–1.77]	0.864	1.92 [1.33–2.75]	<0.001	1.71 [0.72–4.09]	0.226
Master’s degree or above	1.00 [ref]		1.00 [ref]		1.00 [ref]		1.00 [ref]	
Gender by Country								
Male	7.33 [2.61–20.58]	<.001	0.94 [0.47–1.87]	0.860	3.34 [2.34–4.77]	<0.001	3.29 [1.44–7.51]	0.005
Other/prefer not to say	--		--		2.82 [1.55–5.12]	0.001	--	
Female	1.00 [ref]		1.00 [ref]		1.00 [ref]		1.00 [ref]	

*Note*. Variables included were: age, gender, area of residence, and highest education; stratified estimates presented for education and gender because these variables had significant interactions by country; -- results not shown due to small cell size (n < 10).

For gender, only the U.S. had an adequate sample size to show that the “other” or “prefer not to say” gender group had higher odds of very lower trust. In the U.K., there were no significant gender differences found in trust in the adjusted analysis (*p* = 0.860). Males consistently had higher odds of having low trust in the other countries (see Table 3).

## Discussion

The main findings in this study were that respondents that were residents of Norway, compared to those from Australia, the United Kingdom, and the United States, had higher levels of trust in information provided by public authorities after two years of being in a global pandemic. Middle age, male or other genders, living in rural or remote areas, and lower levels of education were significantly associated with very low or low trust, as were gender and education when including interaction terms by country, to test for the robustness of effects by country.

Norway was identified as a country that was immediately responsive and provided daily information through press conferences that were easily accessible to citizens [33, 34]. Outcomes from the Norwegian, but also Australian authority responses that were identified as strong, collaborative, and intentional demonstrate that a response focused on engaging citizens to work collectively can be effective in addressing a global pandemic [33, 35, 36]. In contrast, the U.K. and the U.S. have both been criticized for lack of immediate response to COVID-19 and continue to receive criticism for a delayed response by public authorities as the public quarantine requirements occurred after Norway [37, 38]. The U.K. and the U.S. had a high rate of infections and fatalities, and several researchers reported distrust in the public authority’s responses.

Trust can be associated with the current political climate; therefore, observing differences among countries can support the understanding of factors that impact levels of trust. Political stability, transparency in decision-making, and distribution of access to resources in the U.S. and the U.K. can be a potential explanation for the observed lower levels of trust in responses [9, 23, 24, 35, 39]. Public critiques of the public authorities in leadership in the U.S. and U.K. label leaders as careless and not adhering to global recommendations. The public opinions and critiques of leadership may be contributing factors to the lower trust levels of respondents in these countries. It may be a plausible argument for the odds of reporting very low or low trust, about two times higher in these countries compared to Norway.

The findings provide additional support to adhering to Idowu et al.’s [2] recommendations regarding the social responsibility in the health sector during a global pandemic. Findings from this study also align with examinations of trust among British individuals during COVID-19 [14]. Balaet et al. examined the relationship between feelings of trust, thoughts about COVID-19, and behaviors. To promote public health during a pandemic, it may be imperative to engage with individuals and groups with low levels of trust, as they may be hesitant to engage in prevention strategies recommended by public authorities. Identifying methods of public engagement and trust-building used in countries that have higher rates of trust can provide guidance on ways to improve public trust and compliance. One method of public engagement is to publish data collected directly from participants instead of large datasets collected by public authorities or entities that have lower levels of trust and analyzed by research teams that do not receive funding from public authorities [7, 10, 11, 17–19, 40]. This information can support recent literature reports that trust in the information of public authorities remains a critical factor when addressing a global health crisis [2, 9, 22, 23, 25].

Ongoing efforts to be responsive to people who have low levels of trust in public authorities should be incorporated into policies, procedures, and protocols globally to continue to enhance the safety of individuals and communities.

### Study Limitations

Participants were invited electronically and via social media platforms to participate in our online survey; therefore, those in the population who did not use social media or the internet are not represented in this study. The study included a higher proportion of female respondents and those with higher levels of education. Although anyone with access to the internet within the four countries could participate, we may have a higher number of participants geographically located closer to our landing sites due to recruitment through the universities. The results are, therefore, not representative of the population in the four countries. As the survey was open to an unlimited amount of people, we are unable to report on response rates. The U.S. respondents comprised more than half of the total sample, having a larger influence on the results of the total sample. This study had a small sample of people who identified as other gender (than male or female), which did not reach the sample size required for analysis. Thus, we have a limited understanding of the experience of non-binary respondents. Trust was measured in relation to the COVID-19 pandemic. Since we did not ask about trust in general, we do not have information to compare general levels of trust in the community. There are pre-existing differences in the general levels of trust of the general population between the countries in our study, which may have increased or decreased due to how the government and authorities have responded to the pandemic [41, 42]. Racial identity was not examined as an independent variable concerning trust due to the cultural differences among the participating countries and differences in the interpretation of race. There was no analysis of the types of information received by respondents from public authorities.

### Conclusion

This study examined trust in public authorities held by the general population two years after COVID-19 was declared a global pandemic. We find significant differences in levels of trust between countries and between sociodemographic groups observed.

This study provides input for public authorities on the need to identify and target groups based on their trust level, which may impact their willingness to obtain a vaccine and utilize other prevention strategies, especially regarding the information provided by public officials. It is critical that public authorities use engagement strategies that promote trust in information and policies. Specific consideration to populations that have lower rates of trust should be made along with an understanding of the historical context that impacts engagement.

Practices that increase levels of trust in public authorities implemented by some countries should be expanded to mitigate the ongoing negative impacts of COVID-19 across the globe. Future research needs to continue to measure change over time and focus on shifts in trust in public authorities after the COVID-19 pandemic to consider additional behavioral trends.

Professionals responsible for social welfare, policy development, and program administration should be aware of the differences in the general public’s likelihood to trust information and financial support offerings when developing programs that target diverse population groups. Trust among citizens may be a vital component when seeking to increase the effectiveness of interventions used to support community safety and wellness. Increasing trust may foster higher levels of public health compliance to reduce the spread of COVID-19 and other public health concerns.
